# Supplementary material for: The impact of greenspace or nature-based interventions on cardiovascular health or cancer-related outcomes: A systematic review of experimental studies
Source: PLoS One. 2022 Nov 23;17(11):e0276517. doi: 10.1371/journal.pone.0276517 (PMC9683573; doi:10.1371/journal.pone.0276517)
Supplement: S1 Checklist — (DOCX) [file pone.0276517.s001.docx]

| **Section and Topic** | **Item #** | **Checklist item** | **Location where item is reported** |
| --- | --- | --- | --- |
| **TITLE** | | | Title |
| Title | 1 | Identify the report as a systematic review.  The Impact of greenspace or nature-based interventions on cardiovascular health or cancer related outcomes: A systematic Review of experimental studies | Title |
| **ABSTRACT** | | |  |
| Abstract | 2 | See the PRISMA 2020 for Abstracts checklist.  The title was not identified  Under the background section, the significance of the study was indicated  The objective was indicated.  Methods explained. All details were followed but the synthesis of results not indicated.  Results and discussion were combined with a brief interpretation and implication of the work.  Funding and registration not indicated | Abstract |
| **INTRODUCTION** | | |  |
| Rationale | 3 | Describe the rationale for the review in the context of existing knowledge.  Cardiovascular diseases (CVD) remain the leading cause of global morbidity and mortality while cancer is second (World Health Organization, 2017). CVD and cancer are closely linked in a bidirectional causal relationship due to multiple shared risk factors; and having one of the diseases puts the patient at an increased risk of having the other (Bertero et al., 2019). Each disease affects the treatment of the other, and therefore, has a detrimental impact on outcomes and individual’s quality of life and survival (Bertero et al., 2019).  There is a growing literature evidence on greenspace in improving clinical outcomes through different interventions such as park prescriptions programs, and other nature-based interventions on either cancer or CVD related outcomes (Bikomeye et al., 2021, 2022; Messiah et al., 2016; Müller-Riemenschneider et al., 2018, 2020; Razani et al., 2016). Further some studies have used experimental design; and those studies have not been reviewed thus far.  We reviewed studies that have studied the impact of nature or greenspace interventions (i.e.: green prescriptions, forest bathing, and other nature or greenspace exposures) on two main health outcomes: (1) CVD outcomes, including incidence, prevalence, morbidity and mortality across different kind of CV conditions and (2) cancer outcomes across the cancer control continuum including cancer risk, prevention, detection, diagnosis, treatment, survivorship, end of life or mortality, as well as cancer related quality of life.  The introduction part of the main manuscript has all references and more details. | Introduction |
| Objectives | 4 | Provide an explicit statement of the objective(s) or question(s) the review addresses.  Research question: “What's the Impact of greenspace or nature-based interventions on cardiovascular diseases outcomes or cancer related health outcomes?” | Introduction |
| **METHODS** | | |  |
| Eligibility criteria | 5 | Specify the inclusion and exclusion criteria for the review and how studies were grouped for the syntheses.  The following PICO framework was followed to determine inclusion and exclusion criteria:  P (Population): No restriction  I (Interventions): Exposure to greenspace or NBI  C (Comparison): All types of controls  O (Outcomes): CVD or cancer related only  (1) CVD related outcomes include blood pressure and major adverse cardiovascular outcomes (MACE), as defined in previous studies including occurrence of fatal and nonfatal myocardial infarction, heart failure, cerebrovascular disease or CV accident or stroke (fatal and nonfatal), or coronary artery bypass grafting (CABG) and cardiac arrest.  (2) Cancer related outcomes include lifestyle changes and QoL during cancer survivorship. We used the National Cancer Institute definition of cancer survivorship in defining the cancer survivor's population which proposes that the survivorship period starts the first time the patient was told by a healthcare provider that they have cancer until the end of life.  We considered papers published in English language since we did not have resource to translate other foreign languages.  Table 1 in the main manuscript has a summary of inclusion and exclusion criteria used for this review. | Methods:  Article selection process |
| Information sources | 6 | Specify all databases, registers, websites, organisations, reference lists and other sources searched or consulted to identify studies. Specify the date when each source was last searched or consulted.  Five electronic citation databases (Medline, PsycINFO, Scopus, Web of Science, and Greenfile) were systematically searched on March 10th, 2021. Contact with study authors was planned if some of the information we sought were missing but we found all information we wanted to report and therefore no need for contacting study authors. | Methods |
| Search strategy | 7 | Present the full search strategies for all databases, registers and websites, including any filters and limits used.  A comprehensive literature search was developed in collaboration with a medical librarian and peer reviewed using the Peer Review of Electronic Search Strategies (PRESS) guideline. The full search strategy for all electronic databases is annexed in appendix B in the manuscript.  Below is a sample search strategy for Medline:  Database: Ovid MEDLINE(R) and Epub Ahead of Print, In-Process, In-Data-Review & Other Non-Indexed Citations and Daily <1946 to March 09, 2021>  -------------------------------------------------------------------------------------------------------------------------------------------  1 exp Cardiovascular Diseases/ or exp Cardiovascular System/ or exp Cerebrovascular Disorders/ or exp Rheumatic Heart Disease/ or exp diabetes mellitus/ or Hypercholesteremia/ or exp Dyslipidemias/ (3421210)  2 (cardiomyopath* or peripheral arterial disease* or peripheral artery disease* or Peripheral vascular disease* or Hypercholesterem* or Dyslipidemia* or Arrhythmia* or abnormal heart rhythm* or Heart Valve Disease* or pericardial disease* or Venous Thrombos* or Pulmonary Emboli* or Aortic Valve Stenos* or Pericarditis or Mitral Valve Insufficienc* or Mitral Valve Prolapse*).mp. (477171)  3 (heart* or cardiovascular* or cerebrovascular* or cardio-oncology or MACE or MACEs or coronary artery disease* or stroke* or myocardial infarct* or cardiac arrest* or coronary arterioscleros* or coronary atheroscleros* or brain ischemia* or cerebral hemorrhag* or intracranial hemorrhag* or hypertens* or blood pressur* or diabet* or impaired glucose or prediabet*).mp. (3182153)  4 exp neoplasms/ or (neoplas* or cancer* or tumor* or tumour* or malignan* or oncolog*).mp. (4646529)  5 1 or 2 or 3 or 4 (8650636)  6 ((Greenspace* or green) adj5 (physical activit* or exercise* or gym*)).mp. (306)  7 ((garden or gardens or gardening or greenway or green space* or greenspace* or nature based) adj3 (intervention*  or prescription* or prescrib* or therap*)).mp. (267)  8 (wilderness adj5 (therap* or intervention* or prescription* or prescrib*)).mp. (28)  9 ((nature adj (play or therapy)) or (nature adj3 (prescription* or prescrib*))).mp. (151)  10 ((green or nature or wild or park or parks) adj3 (prescription* or prescrib* or play* or exercis* or gym or gyms  or gymnasium*)).mp. (1787)  11 (forest school* not wake forest).mp. (6)  12 (forest bathing or ecotherap*).mp. (61)  13 6 or 7 or 8 or 9 or 10 or 11 or 12 (2287)  14 5 and 13 (427)  15 14 not (Animals/ not (Animals/ and Humans/)) (372)  16 limit 15 to english language (348)  The above search strategy was translated for other databases; and all strategies are included in Appendix B. | Methods/  Appendix |
| Selection process | 8 | Specify the methods used to decide whether a study met the inclusion criteria of the review, including how many reviewers screened each record and each report retrieved, whether they worked independently, and if applicable, details of automation tools used in the process.  Two independent reviewers (J.C.B. and J.S.B.) collaborated through each phase of the review process from title screening, eligibility, and inclusion. Conflicts were resolved and the process was repeated for full abstract screening, and article’s methods sections. If a conflict could not be resolved between the two reviewers, a third mediator (KMMB) was consulted; and a consensus was reached. | Method |
| Data collection process | 9 | Specify the methods used to collect data from reports, including how many reviewers collected data from each report, whether they worked independently, any processes for obtaining or confirming data from study investigators, and if applicable, details of automation tools used in the process.  During data extraction, we summarized studies methods and findings in two tables and used the following information to create alluvial charts for visual representation of trends across studies by outcomes of interest:  (1) Article reference  (2) Study country  (3) Intervention type  (4) CVD outcomes or cancer-related outcomes.  (5) Conclusion (weather a statistical test was significantly beneficial, or not significant, or no difference between control and experimental groups).  This process was done by J.C.B. and J.S.B. and summary tables for all included studies are in the main manuscripts. Table 3 has CVD outcomes while table 4 has Cancer related outcomes. Alluvial charts were created for (1) specific outcomes: CVD or cancer (Figures 2 and 3) and for (2) specific interventions: forest bathing, green exercise, nature viewing, and gardening (Figures 4 to 7), in the main article.  We initially anticipated requesting specific information from investigators if some of the data needed were missing but found all we need to report and therefore was no need to obtain additional information from investigators. | Method |
| Data items | 10a | List and define all outcomes for which data were sought. Specify whether all results that were compatible with each outcome domain in each study were sought (e.g. for all measures, time points, analyses), and if not, the methods used to decide which results to collect.  All measures of CV health outcomes were considered   1. Systolic blood pressure 2. Diastolic blood pressure 3. Heart rate variability 4. Heart rate/Pulse rate 5. Inflammatory biomarkers 6. Oxidative stress biomarkers 7. Physiological measures of stress markers such as cortisol and adrenaline   For cancer, we considered all survivorship quality of life related measures including   1. Physical activity (PA) 2. Vegetable consumption 3. Health-related quality of life (QoL) 4. Physical performance 5. Immune system measures such as Natural killer (NK) cells activity and/or number of NK cells or T Cells.   All outcomes and how they were measured are summarized in tables 2 and 3 in the main manuscript. | Tables 3 and 4 and Alluvial charts |
|  | 10b | List and define all other variables for which data were sought (e.g. participant and intervention characteristics, funding sources). Describe any assumptions made about any missing or unclear information.  We also extracted information on country of study, sample size, study type, participants characteristics (age) and statistical analyses conducted.  We also extracted data related to greening or nature-based intervention such as forest bathing, nature viewing, green exercise, and vegetable gardening so that we can evaluate the impact of specific types of intervention conducted. | Tables 3 and 4 |
| Study risk of bias assessment | 11 | Specify the methods used to assess risk of bias in the included studies, including details of the tool(s) used, how many reviewers assessed each study and whether they worked independently, and if applicable, details of automation tools used in the process.  A modified version of Newcastle–Ottawa Scale (NOS) was used in the risk of bias assessment. Two reviewers (J.C.B. and J.S.B.) independently assessed articles for eight pre-defined assessment items including representativeness of exposed groups, similarity of groups origins, similarity of exposed vs non-exposed groups (compatibility), ascertainment of exposure, baseline differences, outcome assessment, exposure duration (enough to observe outcome), and groups follow up after greenspace intervention. Table 2 summarizes the risk for bias for all studies. | Methods |
| Effect measures | 12 | Specify for each outcome the effect measure(s) (e.g. risk ratio, mean difference) used in the synthesis or presentation of results.  Depending on outcomes, studies reported means (for example for blood pressure measures). We were interested in whether observed differences were significant or not, and we reported that. | Tables 3 and 4 |
| Synthesis methods | 13a | Describe the processes used to decide which studies were eligible for each synthesis (e.g. tabulating the study intervention characteristics and comparing against the planned groups for each synthesis (item #5)).  A pre-defined PICO criteria was used to decide:  P (Population): No restriction  I (Interventions): Exposure to greenspace or NBI  C (Comparison): All types of controls  O (Outcomes): CVD or cancer related only | Method |
|  | 13b | Describe any methods required to prepare the data for presentation or synthesis, such as handling of missing summary statistics, or data conversions.  We tabulate information into an excel dataset to produce alluvial charts for presentation. |  |
|  | 13c | Describe any methods used to tabulate or visually display results of individual studies and syntheses.  We used alluvial charts to illustrate trends across all studies and all statistical tests conducted by individual studies for all outcome measures. All data used in creating alluvial charts annexed in appendix B in the manuscript. |  |
|  | 13d | Describe any methods used to synthesize results and provide a rationale for the choice(s). If meta-analysis was performed, describe the model(s), method(s) to identify the presence and extent of statistical heterogeneity, and software package(s) used.  We did not conduct a meta-analysis; but we summarized all interventions and outcomes measured in alluvial charts to showcase trend across all statistical tests conducted across individual studies. |  |
|  | 13e | Describe any methods used to explore possible causes of heterogeneity among study results (e.g. subgroup analysis, meta-regression).  Not applicable |  |
|  | 13f | Describe any sensitivity analyses conducted to assess robustness of the synthesized results.  Not applicable |  |
| Reporting bias assessment | 14 | Describe any methods used to assess risk of bias due to missing results in a synthesis (arising from reporting biases).  We used a modified version of the Newcastle–Ottawa Scale (NOS) in conducting the risk of bias assessment. |  |
| Certainty assessment | 15 | Describe any methods used to assess certainty (or confidence) in the body of evidence for an outcome.  Not applicable |  |
| **RESULTS** | | |  |
| Study selection | 16a | Describe the results of the search and selection process, from the number of records identified in the search to the number of studies included in the review, ideally using a flow diagram.  We considered only articles that were published after the rigorous peer-review process with clear measures of outcomes of interests and clear interventions according to our pre-defined PICO. Out of 2,565 articles initially retrieved from database searches, 31 articles meeting our pre-defined criteria remained (PICO); and were included in our review. The PRISMA 2020 chart has all details for this process. | Figure 1: PRISMA 2020 |
|  | 16b | Cite studies that might appear to meet the inclusion criteria, but which were excluded, and explain why they were excluded.  N/A | N/A |
| Study characteristics | 17 | Cite each included study and present its characteristics.  31 studies that fulfilled the PICO criteria and are all cited in table and throughout the paper. | Tables 3 and 4 |
| Risk of bias in studies | 18 | Present assessments of risk of bias for each included study.  A summary score for each study is presented in Table 2. | Table 2 |
| Results of individual studies | 19 | For all outcomes, present, for each study: (a) summary statistics for each group (where appropriate) and (b) an effect estimate and its precision (e.g. confidence/credible interval), ideally using structured tables or plots.  We summarized all individual studies in Tables 3 and 4; and we used alluvial charts to illustrate trends across all studies and all statistical tests conducted by individual studies for all outcome measures. | Tables 3 and 4/ Alluvial charts |
| Results of syntheses | 20a | For each synthesis, briefly summarise the characteristics and risk of bias among contributing studies.  Summarizes were reported by country, by outcomes, by statistical tests, and by outcome measures. | Results |
|  | 20b | Present results of all statistical syntheses conducted. If meta-analysis was done, present for each the summary estimate and its precision (e.g. confidence/credible interval) and measures of statistical heterogeneity. If comparing groups, describe the direction of the effect.  N/A: Neither statistical analysis nor meta-analysis was performed. |  |
|  | 20c | Present results of all investigations of possible causes of heterogeneity among study results.  Not applicable |  |
|  | 20d | Present results of all sensitivity analyses conducted to assess the robustness of the synthesized results.  Not applicable |  |
| Reporting biases | 21 | Present assessments of risk of bias due to missing results (arising from reporting biases) for each synthesis assessed.  Risk of biases are summarized in Table 2 with the modified version of the NOS scale. |  |
| Certainty of evidence | 22 | Present assessments of certainty (or confidence) in the body of evidence for each outcome assessed.  Not applicable |  |
| **DISCUSSION** | | |  |
| Discussion | 23a | Provide a general interpretation of the results in the context of other evidence.  The discussion evolves around the impact of greenspace interventions conduced on outcome measures (CVD or cancer related). Diverse types of exposure to greenspace were identified, including forest bathing, green exercise, vegetable gardening, and nature viewing and assessment of the impact of/or greenspace or NBI on two main health outcomes: (1) CV health, and (2) QoL in cancer survivors. | Discussion |
|  | 23b | Discuss any limitations of the evidence included in the review. | Limitations |
|  | 23c | Discuss any limitations of the review processes used. | Limitations |
|  | 23d | Discuss implications of the results for practice, policy, and future research. | Discussion and Conclusion |
| **OTHER INFORMATION** | | |  |
| Registration and protocol | 24a | Provide registration information for the review, including register name and registration number, or state that the review was not registered.  The protocol for this review is registered with PROSPERO, ID # CRD42021231619. | Methods |
|  | 24b | Indicate where the review protocol can be accessed, or state that a protocol was not prepared.  The protocol for this review is registered with PROSPERO, ID # CRD42021231619 and is accessible online. | Methods |
|  | 24c | Describe and explain any amendments to information provided at registration or in the protocol.  N/A |  |
| Support | 25 | Describe sources of financial or non-financial support for the review, and the role of the funders or sponsors in the review.  The work is supported by an American Heart Association Scientific focused research network on disparities in Cardio-oncology (K.M.M.B. and A.M.B.) grant, NIH (National Institutes of Health) grants: R01HL133029 (A.M.B.), R01CA214805 (K.M.M.B), the Medical College of Wisconsin Cancer Center grants (KM.M.B), and by the We Care Fund (A.M.B.). | Funding |
| Competing interests | 26 | Declare any competing interests of review authors.  All authors have no conflict of interest to disclose | Conflict of interest declaration |
| Availability of data, code and other materials | 27 | Report which of the following are publicly available and where they can be found: template data collection forms; data extracted from included studies; data used for all analyses; analytic code; any other materials used in the review.  Full search strategy and excel dataset used in alluvial charts are included in Appendices. | Appendix B and C |

*From:*  Page MJ, McKenzie JE, Bossuyt PM, Boutron I, Hoffmann TC, Mulrow CD, et al. The PRISMA 2020 statement: an updated guideline for reporting systematic reviews. BMJ 2021;372:n71. doi: 10.1136/bmj.n71

For more information, visit: <http://www.prisma-statement.org/>
