# Supplementary material for: The impact of greenspace or nature-based interventions on cardiovascular health or cancer-related outcomes: A systematic review of experimental studies
Source: PLoS One. 2022 Nov 23;17(11):e0276517. doi: 10.1371/journal.pone.0276517 (PMC9683573; doi:10.1371/journal.pone.0276517)
Supplement: S1 File — (DOCX) [file pone.0276517.s002.docx]

# Supporting Information

**APPENDICES**

1. **PRISMA 2020 Checklist**

The PRISMA 2020 Checklist is attached as a separate file to this manuscript submission.

1. **Full Search Strategy**

Database: Ovid MEDLINE(R) and Epub Ahead of Print, In-Process, In-Data-Review & Other Non-Indexed Citations and Daily <1946 to March 09, 2021>

-------------------------------------------------------------------------------------------------------------------------------------------

1 exp Cardiovascular Diseases/ or exp Cardiovascular System/ or exp Cerebrovascular Disorders/ or exp Rheumatic Heart Disease/ or exp diabetes mellitus/ or Hypercholesteremia/ or exp Dyslipidemias/ (3421210)

2 (cardiomyopath* or peripheral arterial disease* or peripheral artery disease* or Peripheral vascular disease* or Hypercholesterem* or Dyslipidemia* or Arrhythmia* or abnormal heart rhythm* or Heart Valve Disease* or pericardial disease* or Venous Thrombos* or Pulmonary Emboli* or Aortic Valve Stenos* or Pericarditis or Mitral Valve Insufficienc* or Mitral Valve Prolapse*).mp. (477171)

3 (heart* or cardiovascular* or cerebrovascular* or cardio-oncology or MACE or MACEs or coronary artery disease* or stroke* or myocardial infarct* or cardiac arrest* or coronary arterioscleros* or coronary atheroscleros* or brain ischemia* or cerebral hemorrhag* or intracranial hemorrhag* or hypertens* or blood pressur* or diabet* or impaired glucose or prediabet*).mp. (3182153)

4 exp neoplasms/ or (neoplas* or cancer* or tumor* or tumour* or malignan* or oncolog*).mp. (4646529)

5 1 or 2 or 3 or 4 (8650636)

6 ((Greenspace* or green) adj5 (physical activit* or exercise* or gym*)).mp. (306)

7 ((garden or gardens or gardening or greenway or green space* or greenspace* or nature based) adj3 (intervention*

or prescription* or prescrib* or therap*)).mp. (267)

8 (wilderness adj5 (therap* or intervention* or prescription* or prescrib*)).mp. (28)

9 ((nature adj (play or therapy)) or (nature adj3 (prescription* or prescrib*))).mp. (151)

10 ((green or nature or wild or park or parks) adj3 (prescription* or prescrib* or play* or exercis* or gym or gyms

or gymnasium*)).mp. (1787)

11 (forest school* not wake forest).mp. (6)

12 (forest bathing or ecotherap*).mp. (61)

13 6 or 7 or 8 or 9 or 10 or 11 or 12 (2287)

14 5 and 13 (427)

15 14 not (Animals/ not (Animals/ and Humans/)) (372)

16 limit 15 to english language (348)

*************************************************************************************************************

Database: APA PsycINFO <1806 to March Week 1 2021>

1 exp Cardiovascular Disorders/ or exp Cardiovascular System/ or exp Cerebrovascular Disorders/ or exp Rheumatic Heart Disease/ or exp diabetes mellitus/ or Hypercholesteremia/ or exp Dyslipidemias/ (79013)

2 (cardiomyopath* or peripheral arterial disease* or peripheral artery disease* or Peripheral vascular disease* or Hypercholesterem* or Dyslipidemia* or Arrhythmia* or abnormal heart rhythm* or Heart Valve Disease* or pericardial disease* or Venous Thrombos* or Pulmonary Emboli* or Aortic Valve Stenos* or Pericarditis or Mitral Valve Insufficienc* or Mitral Valve Prolapse*).mp. (7179)

3 (heart* or cardiovascular* or cerebrovascular* or cardio-oncology or MACE or MACEs or coronary artery disease* or stroke* or myocardial infarct* or cardiac arrest* or coronary arterioscleros* or coronary atheroscleros* or brain ischemia* or cerebral hemorrhag* or intracranial hemorrhag* or hypertens* or blood pressur* or diabet* or impaired glucose or prediabet*).mp. (185372)

4 exp neoplasms/ or (neoplas* or cancer* or tumor* or tumour* or malignan* or oncolog*).mp. (91702)

5 1 or 2 or 3 or 4 (275122)

6 ((Greenspace* or green) adj5 (physical activit* or exercise* or gym*)).mp. (83)

7 ((garden or gardens or gardening or greenway or green space* or greenspace* or nature based) adj3 (intervention*

or prescription* or prescrib* or therap*)).mp. (174)

8 (wilderness adj5 (therap* or intervention* or prescription* or prescrib*)).mp. (218)

9 ((nature adj (play or therapy)) or (nature adj3 (prescription* or prescrib*))).mp. (103)

10 ((green or nature or wild or park or parks) adj3 (prescription* or prescrib* or play* or exercis* or gym or gyms

or gymnasium*)).mp. (913)

11 (forest school* not wake forest).mp. (44)

12 (forest bathing or ecotherap*).mp. (68)

13 6 or 7 or 8 or 9 or 10 or 11 or 12 (1443)

14 5 and 13 (71)

15 limit 14 to english language (68)

*************************************************************************************************************

Scopus

( ( TITLE-ABS-KEY ( ( greenspace* OR green ) W/5 ( "physical activit*" OR exercise* OR gym* ) ) ) OR ( TITLE-ABS-KEY ( ( garden OR gardens OR gardening OR greenway OR "green space*" OR greenspace* OR "nature based" ) W/3 ( intervention* OR prescription* OR prescrib* OR therap* ) ) ) OR ( TITLE-ABS-KEY ( ( wilderness W/5 ( therap* OR intervention* OR prescription* OR prescrib* ) ) ) ) OR ( TITLE-ABS-KEY ( "nature play" OR "nature therapy" OR ( nature W/3 ( prescrib* OR prescription* ) ) ) ) OR ( TITLE-ABS-KEY ( ( ( green OR nature OR wild OR park OR parks ) W/3 ( prescription* OR prescrib* OR play* OR exercis* OR gym OR gyms OR gymnasium* ) ) ) ) OR ( TITLE-ABS-KEY ( ( "forest school*" AND NOT "wake forest" ) ) ) OR ( TITLE-ABS-KEY ( ( "forest bathing" OR ecotherap* ) ) ) ) AND ( TITLE-ABS-KEY ( ( cardiomyopath* OR "peripheral arterial disease*" OR "peripheral artery disease*" OR "Peripheral vascular disease*" OR hypercholesterem* OR dyslipidemia* OR arrhythmia* OR "abnormal heart rhythm*" OR "Heart Valve Disease*" OR "pericardial disease*" OR "Venous Thrombos*" OR "Pulmonary Emboli*" OR "Aortic Valve Stenos*" OR pericarditis OR "Mitral Valve Insufficienc*" OR "Mitral Valve Prolapse*" OR heart* OR cardiovascular* OR cerebrovascular* OR "cardio-oncology" OR mace OR maces OR "coronary artery disease*" OR stroke* OR "myocardial infarct*" OR "cardiac arrest*" OR "coronary arterioscleros*" OR "coronary atheroscleros*" OR "brain ischemia*" OR "cerebral hemorrhag*" OR "intracranial hemorrhag*" OR hypertens* OR "blood pressur*" OR diabet* OR "impaired glucose" OR prediabet* OR neoplas* OR cancer* OR tumor* OR tumour* OR malignan* OR oncolog* ) ) ) AND ( LIMIT-TO ( LANGUAGE , "English" ) )

1161 result

*************************************************************************************************************

Web of Science

(Databases= WOS Core Collection, BCI Biosis Citation Index, CCC Current Contents Connect, DRCI Data Citation Index, DIIDW Derwent Innovations Index, KJD Korean Journakl Database, MEDLINE, RSCI Russian Science Citation Index, SCIELO ScieELO Citation Index, ZOOREC Zoological Record)

Timespan=All years

Search language=English

# 11

972

#9 AND #1

Refined by: LANGUAGES: ( ENGLISH )

# 10

1,068

#9 AND #1

# 9

11,839

#8 OR #7 OR #6 OR #5 OR #4 OR #3 OR #2

# 8

190

TS=( ( "forest bathing" OR ecotherap* ) )

# 7

147

TS=( ( "forest school*" NOT "wake forest" ) )

# 6

10,305

TS=( ( ( green OR nature OR wild OR park OR parks ) NEAR/3 ( prescription* OR prescrib* OR play* OR exercis* OR gym OR gyms OR gymnasium* ) ) )

# 5

587

TS=( "nature play" OR "nature therapy" OR ( nature NEAR/3 ( prescrib* OR prescription* ) ) )

# 4

137

TS=( ( wilderness NEAR/5 ( therap* OR intervention* OR prescription* OR prescrib* ) ) )

# 3

657

TS=( ( garden OR gardens OR gardening OR greenway OR "green space*" OR greenspace* OR "nature based" ) NEAR/3 ( intervention* OR prescription* OR prescrib* OR therap* ) )

# 2

862

TS=( ( greenspace* OR green ) NEAR/5 ( "physical activit*" OR exercise* OR gym* ) )

# 1

14,956,179

TS=( ( cardiomyopath* OR "peripheral arterial disease*" OR "peripheral artery disease*" OR "Peripheral vascular disease*" OR hypercholesterem* OR dyslipidemia* OR arrhythmia* OR "abnormal heart rhythm*" OR "Heart Valve Disease*" OR "pericardial disease*" OR "Venous Thrombos*" OR "Pulmonary Emboli*" OR "Aortic Valve Stenos*" OR pericarditis OR "Mitral Valve Insufficienc*" OR "Mitral Valve Prolapse*" OR heart* OR cardiovascular* OR cerebrovascular* OR "cardio-oncology" OR mace OR maces OR "coronary artery disease*" OR stroke* OR "myocardial infarct*" OR "cardiac arrest*" OR "coronary arterioscleros*" OR "coronary atheroscleros*" OR "brain ischemia*" OR "cerebral hemorrhag*" OR "intracranial hemorrhag*" OR hypertens* OR "high blood pressur*" OR diabet* OR "impaired glucose" OR prediabet* OR neoplas* OR cancer* OR tumor* OR tumour* OR malignan* OR oncolog* ) )

*************************************************************************************************************

GREENFILE

S12 S3 AND S11

Database - GreenFILE 16

S11 S4 OR S5 OR S6 OR S7 OR S8 OR S9 OR S10

Database - GreenFILE 580

S10 ( ( "forest bathing" OR ecotherap* ) )

Database - GreenFILE 9

S9 ( ( "forest school*" NOT "wake forest" ) )

Database - GreenFILE 63

S8 ( ( ( green OR nature OR wild OR park OR parks ) N3 ( prescription* OR prescrib* OR play* OR exercis* OR gym OR gyms OR gymnasium* ) ) )

Database - GreenFILE 415

S7 ( "nature play" OR "nature therapy" OR ( nature N3 ( prescrib* OR prescription* ) ) )

Database - GreenFILE 19

S6 ( ( wilderness N5 ( therap* OR intervention* OR prescription* OR prescrib* ) ) )

Database - GreenFILE 7

S5 ( ( garden OR gardens OR gardening OR greenway OR "green space*" OR greenspace* OR "nature based" ) N3 ( intervention* OR prescription* OR prescrib* OR therap* ) )

Database - GreenFILE 55

S4 ( greenspace* OR green ) N5 ( "physical activit*" OR exercise* OR gym* )

Database - GreenFILE 63

S3 S1 OR S2

Database - GreenFILE 22,680

S2 cardiovascular* OR cerebrovascular* OR "cardio-oncology" OR mace OR maces OR "coronary artery disease*" OR stroke* OR "myocardial infarct*" OR "cardiac arrest*" OR "coronary arterioscleros*" OR "coronary atheroscleros*" OR "brain ischemia*" OR "cerebral hemorrhag*" OR "intracranial hemorrhag*" OR hypertens* OR "high blood pressur*" OR diabet* OR "impaired glucose" OR prediabet* OR neoplas* OR cancer* OR tumor* OR tumour* OR malignan* OR oncolog*

Database - GreenFILE 19,055

S1 cardiomyopath* OR "peripheral arterial disease*" OR "peripheral artery disease*" OR "Peripheral vascular disease*" OR hypercholesterem* OR dyslipidemia* OR arrhythmia* OR "abnormal heart rhythm*" OR "Heart Valve Disease*" OR "pericardial disease*" OR "Venous Thrombos*" OR "Pulmonary Emboli*" OR "Aortic Valve Stenos*" OR pericarditis OR "Mitral Valve Insufficienc*" OR "Mitral Valve Prolapse*" OR heart*

Database - GreenFILE 5,053

**C. Excel datasets used in Alluvial charts**

**C.1. Cardiovascular health related outcomes**

| **ARTICLES** | **COUNTRIES** | **INTERVENTIONS** | **OUTCOMES** | **CONCLUSION** |
| --- | --- | --- | --- | --- |
| Bielinis et al., 2019 | Poland | Forest bathing | SBP | Beneficial effect |
| Bielinis et al., 2019 | Poland | Forest bathing | DBP | Not significant |
| Bielinis et al., 2019 | Poland | Forest bathing | HR | Beneficial effect |
| Chen et al., 2018 | Taiwan | Forest bathing | SBP | Beneficial effect |
| Chen et al., 2018 | Taiwan | Forest bathing | DBP | Not significant |
| Chen et al., 2018 | Taiwan | Forest bathing | HR | Not significant |
| Duncan et al., 2014 | UK | Green exercise | SBP | Beneficial effect |
| Duncan et al., 2014 | UK | Green exercise | DBP | Not significant |
| Duncan et al., 2014 | UK | Green exercise | HR | Not significant |
| Engell et al., 2020 | Norway | Nature viewing | HR | Beneficial effect |
| Furuyashiki et al., 2019 | Japan | Forest bathing | SBP | Beneficial effect |
| Furuyashiki et al., 2019 | Japan | Forest bathing | DBP | Beneficial effect |
| Furuyashiki et al., 2019 | Japan | Forest bathing | HR | Not significant |
| Grazuleviciene et al., 2016 | Lithuania | Green exercise | DBP | Beneficial effect |
| Grazuleviciene et al., 2016 | Lithuania | Green exercise | SBP | Not significant |
| Koura et al., 2016 | Japan | Gardening | PNSA | Beneficial effect |
| Koura et al., 2016 | Japan | Gardening | SNSA | Beneficial effect |
| Lanki et al., 2017 | Finland | Green exercise | SBP | Not significant |
| Lanki et al., 2017 | Finland | Green exercise | DBP | Not significant |
| Lanki et al., 2017 | Finland | Green exercise | HR | Beneficial effect |
| Lanki et al., 2017 | Finland | Green exercise | HRV | Beneficial effect |
| Lee et al. 2011 | Japan | Forest bathing | PNSA | Beneficial effect |
| Lee et al. 2011 | Japan | Forest bathing | SNSA | Beneficial effect |
| Lee et al. 2011 | Japan | Forest bathing | HR | Beneficial effect |
| Li et al., 2016 | Japan | Forest bathing | HR | Beneficial effect |
| Li et al., 2016 | Japan | Forest bathing | SBP | Not significant |
| Li et al., 2016 | Japan | Forest bathing | DBP | Not significant |
| Li et al., 2016 | Japan | Forest bathing | AdipoQ | Beneficial effect |
| Mao et al., 2012 | China | Forest bathing | SBP | Beneficial effect |
| Mao et al., 2012 | China | Forest bathing | DBP | Beneficial effect |
| Mao et al., 2012 | China | Forest bathing | HR | Not significant |
| Mao et al., 2012 | China | Forest bathing | Hcy | Beneficial effect |
| Mao et al., 2012 | China | Forest bathing | PP | Not significant |
| Mao et al., 2012 | China | Forest bathing | ET-1 | Beneficial effect |
| Mao et al., 2012 | China | Forest bathing | RAS | Beneficial effect |
| Mao et al., 2012 (2) | China | Forest bathing | ET-1 | Beneficial effect |
| Mao et al., 2012 (2) | China | Forest bathing | MDA | Beneficial effect |
| Mao et al., 2012 (2) | China | Forest bathing | TNF-α | Beneficial effect |
| Mao et al., 2017 | China | Forest bathing | BNP | Beneficial effect |
| Mao et al., 2017 | China | Forest bathing | ET-1 | Beneficial effect |
| Mao et al., 2017 | China | Forest bathing | RAS | Beneficial effect |
| McEwan et al., 2021 | UK | Forest bathing | HRV | Beneficial effect |
| Navalta et al., 2021 | US | Green exercise | HR | Not significant |
| Navalta et al., 2021 | US | Green exercise | SBP | Not significant |
| Navalta et al., 2021 | US | Green exercise | DBP | Not significant |
| Niedermeier et al., 2017 | Austria | Green exercise | SBP | Not significant |
| Niedermeier et al., 2017 | Austria | Green exercise | DBP | Not significant |
| Niedermeier et al., 2017 | Austria | Green exercise | HR | Not significant |
| Ochiai et al., 2015 | Japan | Forest bathing | SBP | Beneficial effect |
| Ochiai et al., 2015 | Japan | Forest bathing | DBP | Beneficial effect |
| Park et al., 2017 | South Korea | Gardening | HDL | Beneficial effect |
| Park et al., 2017 | South Korea | Gardening | SBP | Beneficial effect |
| Park et al., 2017 | South Korea | Gardening | DBP | Beneficial effect |
| Park et al., 2017 | South Korea | Gardening | LDL | Not significant |
| Park et al., 2017 | South Korea | Gardening | iNOS | Not significant |
| Park et al., 2017 | South Korea | Gardening | RAGE | Beneficial effect |
| Park et al., 2017 | South Korea | Gardening | NADPH | Not significant |
| Park et al., 2017 | South Korea | Gardening | TNF-α | Beneficial effect |
| Park et al., 2017 | South Korea | Gardening | MCP-1 | Not significant |
| Peterfalvi et al., 2021 | Hungary | Forest bathing | SBP | Beneficial effect |
| Peterfalvi et al., 2021 | Hungary | Forest bathing | DBP | Not significant |
| Peterfalvi et al., 2021 | Hungary | Forest bathing | HR | Not significant |
| Pretty et al., 2005 | UK | Green exercise | SBP | Beneficial effect |
| Pretty et al., 2005 | UK | Green exercise | DBP | Beneficial effect |
| Pretty et al., 2005 | UK | Green exercise | HR | Not significant |
| Song et al., 2013 | Japan | Green exercise | HR | Beneficial effect |
| Song et al., 2013 | Japan | Green exercise | HRV | Beneficial effect |
| Song et al., 2018 | Japan | Nature viewing | PNSA | Beneficial effect |
| Song et al., 2018 | Japan | Nature viewing | SNSA | Beneficial effect |
| Song et al., 2018 | Japan | Nature viewing | HR | Not significant |
| Tsutsumi et al., 2017 | Japan | Nature viewing | HR | Beneficial effect |
| Tsutsumi et al., 2017 | Japan | Nature viewing | SBP | Not significant |
| Tsutsumi et al., 2017 | Japan | Nature viewing | DBP | Not significant |
| Tsutsumi et al., 2017 | Japan | Nature viewing | PNSA | Beneficial effect |
| White et al., 2015 | UK | Green exercise | SBP | Beneficial effect |
| White et al., 2015 | UK | Green exercise | DBP | Beneficial effect |
| White et al., 2015 | UK | Green exercise | HR | Beneficial effect |
| Wu et al., 2020 | China | Forest bathing | SBP | Not significant |
| Wu et al., 2020 | China | Forest bathing | DBP | Beneficial effect |
| Wu et al., 2020 | China | Forest bathing | HR | Not significant |
| Wu et al., 2020 | China | Forest bathing | HRV | Beneficial effect |
| Wu et al., 2020 | China | Forest bathing | hsCRP | Beneficial effect |
| Yu et al., 2017 | Taiwan | Forest bathing | SBP | Beneficial effect |
| Yu et al., 2017 | Taiwan | Forest bathing | DBP | Beneficial effect |
| Yu et al., 2017 | Taiwan | Forest bathing | HR | Beneficial effect |
| Yu et al., 2017 | Taiwan | Forest bathing | HRV | Not significant |
| Yu et al., 2017 | Taiwan | Forest bathing | PNSA | Not significant |
| Yu et al., 2017 | Taiwan | Forest bathing | SNSA | Not significant |

*Acronyms*: SBP^1^: Systolic blood pressure; DBP^2^: Diastolic blood pressure; BNP^3^: Brain natriuretic peptide; HRV^4^: Heart rate variability; RAS^5^: Renin-angiotensin system components; PNSA^6^: Parasympathetic Nervous System Activity; SNSN^7^: Sympathetic Nervous System Activity; hsCRP^8^: High sensitivity C-reactive protein, TNF- α^9^: Tumor necrosis factor alpha, HR^10^: Heart rate, MDA^11^: Malondialdehyde, RAGE^12^: Receptor for advanced glycation end products, iNOS^13^ Inducible nitric oxide synthase, MCP-1^14^: Monocyte chemoattractant protein-1, ET-1^15^: Endothelin-1, PP^16^: Pulse pressure, AdipoQ^17^: Adiponectin, Hcy^18^: Homocysteine

**C.2: Cancer-related outcomes**

| **ARTICLES** | **COUNTRIES** | **INTERVENTIONS** | **OUTCOMES** | **CONCLUSION** |
| --- | --- | --- | --- | --- |
| Bail et al., 2018 | US | Gardening | Vegetable consumption | Beneficial effect |
| Bail et al., 2018 | US | Gardening | Improved PA | Beneficial effect |
| Bail et al., 2018 | US | Gardening | 2-minute-step test | Beneficial effect |
| Bail et al., 2018 | US | Gardening | Gardening continuation | Beneficial effect |
| Bail et al., 2018 | US | Gardening | Telomerase activity | Significant in controls |
| Blair et al., 2013 | US | Gardening | Improved strength | Beneficial effect |
| Blair et al., 2013 | US | Gardening | Improved endurance | Beneficial effect |
| Blair et al., 2013 | US | Gardening | Improved PA | Beneficial effect |
| Blair et al., 2013 | US | Gardening | Vegetable consumption | Beneficial effect |
| Blair et al., 2013 | US | Gardening | Fruits consumption | Beneficial effect |
| Blair et al., 2013 | US | Gardening | Improved agility | Beneficial effect |
| Blair et al., 2013 | US | Gardening | Weight loss | Not significant |
| Blair et al., 2013 | US | Gardening | Overall QoL | Not significant |
| Demark-Wahnefried et al., 2018 | US | Gardening | Reassurance of worth | Beneficial effect |
| Demark-Wahnefried et al., 2018 | US | Gardening | Fruits consumption | Beneficial effect |
| Demark-Wahnefried et al., 2018 | US | Gardening | Vegetable consumption | Beneficial effect |
| Demark-Wahnefried et al., 2018 | US | Gardening | Telomerase activity | Beneficial effect |
| Demark-Wahnefried et al., 2018 | US | Gardening | Cortisol | Not significant |
| Demark-Wahnefried et al., 2018 | US | Gardening | IL-6 | Not significant |
| Demark-Wahnefried et al., 2018 | US | Gardening | Overall QoL | Significant in controls |
| Li et al., 2008 | Japan | Forest bathing | Increased NK activity | Beneficial effect |
| Li et al., 2008 | Japan | Forest bathing | Increased # of NK | Beneficial effect |
| Li et al., 2007 | Japan | Forest bathing | Increased NK activity | Beneficial effect |
| Li et al., 2007 | Japan | Forest bathing | Increased # of NK | Beneficial effect |

*Acronyms*: PA^1^: Physical activity; NK^2^: Natural killer cells; QoL^3^: Quality of life; and IL-6^4^: Interleukin-6.
